# Loss of KCC2 in GABAergic Neurons Causes Seizures and an Imbalance of Cortical Interneurons

**DOI:** 10.3389/fnmol.2022.826427

**Published:** 2022-03-16

**Authors:** Kirill Zavalin, Anjana Hassan, Cary Fu, Eric Delpire, Andre H. Lagrange

**Affiliations:** ^1^Department of Neurology, School of Medicine, Vanderbilt University, Nashville, TN, United States; ^2^Department of Pediatrics, Vanderbilt University Medical Center, Nashville, TN, United States; ^3^Department of Anesthesiology, School of Medicine, Vanderbilt University, Nashville, TN, United States; ^4^Department of Neurology, Tennessee Valley Healthcare – Veterans Affairs (TVH VA), Medical Center, Nashville, TN, United States

**Keywords:** KCC2, interneuron, excitatory GABA, excitatory/inhibitory balance, development, somatostatin interneuron, parvalbumin interneuron, seizure

## Abstract

K-Cl transporter KCC2 is an important regulator of neuronal development and neuronal function at maturity. Through its canonical transporter role, KCC2 maintains inhibitory responses mediated by γ-aminobutyric acid (GABA) type A receptors. During development, late onset of KCC2 transporter activity defines the period when depolarizing GABAergic signals promote a wealth of developmental processes. In addition to its transporter function, KCC2 directly interacts with a number of proteins to regulate dendritic spine formation, cell survival, synaptic plasticity, neuronal excitability, and other processes. Either overexpression or loss of KCC2 can lead to abnormal circuit formation, seizures, or even perinatal death. GABA has been reported to be especially important for driving migration and development of cortical interneurons (IN), and we hypothesized that properly timed onset of KCC2 expression is vital to this process. To test this hypothesis, we created a mouse with conditional knockout of KCC2 in Dlx5-lineage neurons (Dlx5 KCC2 cKO), which targets INs and other post-mitotic GABAergic neurons in the forebrain starting during embryonic development. While KCC2 was first expressed in the INs of layer 5 cortex, perinatal IN migrations and laminar localization appeared to be unaffected by the loss of KCC2. Nonetheless, the mice had early seizures, failure to thrive, and premature death in the second and third weeks of life. At this age, we found an underlying change in IN distribution, including an excess number of somatostatin neurons in layer 5 and a decrease in parvalbumin-expressing neurons in layer 2/3 and layer 6. Our research suggests that while KCC2 expression may not be entirely necessary for early IN migration, loss of KCC2 causes an imbalance in cortical interneuron subtypes, seizures, and early death. More work will be needed to define the specific cellular basis for these findings, including whether they are due to abnormal circuit formation versus the sequela of defective IN inhibition.

## Introduction

For over a decade, K-Cl transporter KCC2 has been in a spotlight of attention due to multiple important roles it plays in regulation of CNS development and neuronal function in adult CNS. This protein serves a number of functions, including cytoskeletal dynamics, dendrite regulation, and ion channel trafficking. However, by far the most widely published function of KCC2 is its “canonical” role to help regulate the neuronal membrane Cl^–^ gradient, which in turn maintains inhibitory responses to γ-aminobutyric acid (GABA). Mutations that impair KCC2 function are associated with schizophrenia, autism spectrum disorders (Merner et al., 2015), and several seizure types (Kahle et al., 2014; Saitsu et al., 2016; Saito et al., 2017) in human patients. In rodents, full KCC2 knockout animals die at birth (Hübner et al., 2001), and knockout of one of two isoforms, KCC2b, dies in the third postnatal week from seizures (Woo et al., 2002). Moreover, mutations in KCC2 that impair its ability to extrude Cl^–^ without otherwise affecting expression likewise result in epilepsy phenotypes (Watanabe et al., 2019).

A prominent hypothesis in the field is that KCC2 prevents seizures by maintaining inhibitory responses through GABA type A receptors (GABARs) (Huberfeld et al., 2007; Kahle et al., 2008; Silayeva et al., 2015). GABARs are pentameric ligand-gated Cl^–^ channels and the primary source of inhibitory neurotransmission in the adult CNS (Engin et al., 2018). Activation of GABARs inhibits neurons by hyperpolarizing the neuronal membrane due to an influx of Cl^–^, which relies on a low neuronal [Cl^–^]. KCC2 transporter activity helps maintain this low neuronal [Cl^–^] (Rivera et al., 1999; Zhu et al., 2005; Di Cristo et al., 2018; Akita and Fukuda, 2020; Otsu et al., 2020), though the importance of KCC2 in setting the neuronal [Cl^–^] has been debated in favor of alternative mechanisms, such as distribution of impermeable anionic charges (Delpire and Staley, 2014; Glykys et al., 2017). Nonetheless, KCC2 is vital in facilitating Cl^–^ transport to this setpoint and maintaining low internal [Cl^–^]. Elimination of KCC2 in neurons results in higher intracellular [Cl^–^] and inability to rapidly extrude Cl^–^ upon Cl^–^ loading, such as during prolonged periods of activity (Di Cristo et al., 2018; Dzhala and Staley, 2021).

In the developing brain, GABA acts contrastingly to its function in adult CNS by stimulating multiple processes of neuronal development (Platel et al., 2010; Luhmann et al., 2015; Wu and Sun, 2015). Immature neurons do not strongly express KCC2, but express a Na-K-Cl transporter NKCC1, which increases internal [Cl^–^], often resulting in depolarizing responses that increase neuronal excitability and may elicit intracellular calcium transients (Kaila et al., 2014; Schulte et al., 2018), though whether GABAergic depolarization is always able to directly excite developing neurons *in vivo* remains a matter of debate (Kirmse et al., 2015; Valeeva et al., 2016; Murata and Colonnese, 2020; Kilb, 2021; Ben-Ari and Cherubini, 2022). This increased excitability caused by altered Cl^–^ homeostasis has been shown to be necessary for many of the developmental processes driven by GABA (Ge et al., 2006; Cancedda et al., 2007; Allene et al., 2008; Bortone and Polleux, 2009; Inada et al., 2011; Young et al., 2012; Griguoli and Cherubini, 2017; Fukuda, 2020; Peerboom and Wierenga, 2021).

Studies show that onset of KCC2 expression is a regulator of neuronal development (Dehorter et al., 2012; Llano et al., 2020; Peerboom and Wierenga, 2021), which is likely mediated by both transporter and transport-independent functions. For instance, the onset of KCC2 expression coincides with the transition to inhibitory GABA responses (Dehorter et al., 2012; Fukuda, 2020; Murata and Colonnese, 2020; Kilb, 2021) and is tightly coupled to precede emergence of GABAergic synaptic activity (Kobayashi et al., 2008). At the same time, this point marks the cessation of developmental programs driven by depolarizing GABA at appropriate postnatal timepoints. Independently of its transporter function, KCC2 facilitates a number of processes that are central to circuit development, such as actin re-arrangement/formation of dendritic spines and plasticity of glutamatergic synapses (Llano et al., 2020), and may be an anti-apoptotic factor regulating neuronal survival (Horn et al., 2010; Mavrovic et al., 2020; Virtanen et al., 2021).

Several groups have proposed that migration and possibly other developmental processes in cortical interneurons (INs) are terminated by KCC2 expression (Bortone and Polleux, 2009; Inamura et al., 2012; Zechel et al., 2016; Peerboom and Wierenga, 2021). Though INs comprise only ∼20% of cortical neurons, they are the only GABAergic neurons in cortex, and are critical to regulating activity of cortical circuits through a variety of mechanisms (Takada et al., 2014). This diversity of inhibitory signaling is mediated by a number of functionally distinct subtypes, such as parvalbumin (PV) and somatostatin (SST) INs, which have different birth origins (Wonders and Anderson, 2006; Lim et al., 2018) and properties at maturity (Markram et al., 2004; Kepecs and Fishell, 2014; Williams and Riedemann, 2021). Deficits in IN development and function underlie neurologic and psychiatric disorders, such as epilepsy (Bozzi et al., 2012; Righes Marafiga et al., 2021), schizophrenia, bipolar disorder, and autism spectrum disorders (Rossignol, 2011; Marin, 2012; Ruden et al., 2021).

The INs are born in subpallial ganglionic eminences and rely on GABAergic signaling to migrate through striatum to cortex, and then tangentially along cortical migratory zones before settling throughout the cortical layers. Drugs that modulate GABARs or disrupt the Cl^–^ electrochemical gradient interfere with IN migration (Cuzon et al., 2006; Bortone and Polleux, 2009; Inada et al., 2011). INs are vital to some forms of nascent cortical network activity, including “giant depolarizing potentials” *ex vivo* (Allene et al., 2008), which have been shown to help incorporate neurons into functional circuits in hippocampal studies (Griguoli and Cherubini, 2017). These observations compound with the aforementioned developmental studies in other neuronal types, indicating that KCC2 expression may be crucial to regulating multiple aspects of IN development, but requires further examination *in vivo*.

We hypothesized that well-timed expression of KCC2 is vital for regulating IN development. Indeed, we found KCC2 is expressed remarkably early in life in layer 5 cortical INs. To test our hypothesis, we created a conditional knockout mouse that lacks KCC2 in INs (Dlx5 KCC2 cKO). Contrary to our initial hypothesis, we found no major differences in perinatal migration or laminar localization of INs in KCC2 KO mice. Nonetheless, the mice had early seizures, failure to thrive, and premature death similar to a full KCC2b knockout. We found that KCC2 disruption produced a layer-specific imbalance of IN subtypes. We hypothesize that together with defective synaptic inhibition, this imbalance contributes to Dlx5:KCC2 cKO pathology.

## Results

### Cortical Interneurons Precociously Express KCC2

**FIGURE 1:**
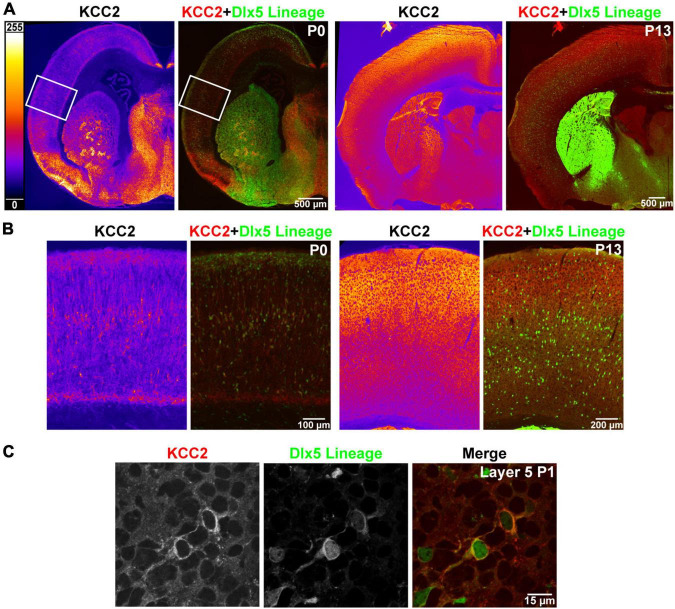
Precocious expression of KCC2 in layer 5 INs. **(A)** KCC2 immunoreactivity in P0 and P13 WT cortex (orange heatmap images) and its overlay with Dlx5:cre tdTomato reporter, which marks cortical interneurons (KCC2 red, Dlx5 reporter green). **(B)** Magnified areas of P0 cortex indicated by a white rectangle in **(A)** and matching images of barrel field cortex at P13. **(C)** Confocal images from layer 5 showing plasmalemmal KCC2 expression in two WT cells expressing a Dlx5 GFP reporter.

Like previous reports, we found that strong cortical expression of KCC2 only begins after the first postnatal week of life. Expression of KCC2 is nearly absent in perinatal cortex, and intermediate and marginal zone, being restricted mainly to a select number of cells within layer 5. To identify these cells, we used transgenic Dlx5:cre-IRES-EGFP mice, where the Dlx5 promoter drives cre and fluorescent GFP reporter expression exclusively in GABAergic neurons within forebrain. Because many INs lose Dlx5 expression with maturity (data not shown), we additionally used the Ai14 cre-inducible tdTomato reporter for permanent labeling. We collected cortical brain sections of P0 mice expressing these reporters and stained them with the KCC2 antibody. We found that at this timepoint, INs are the only cells in cortex that express KCC2, as plasmalemmal KCC2 immunoreactivity was only seen in cells with somatic expression of the tdTomato reporter (Figure 1).

**FIGURE 2:**
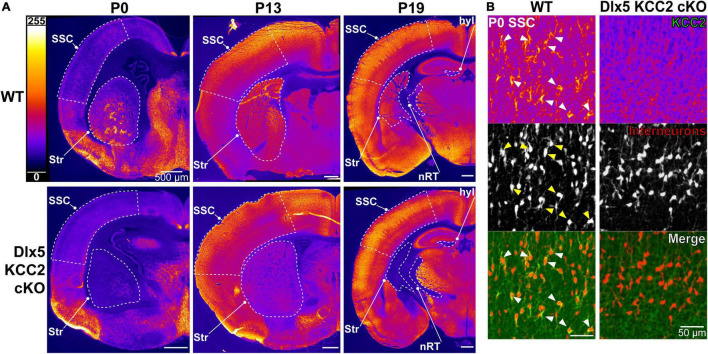
Loss of KCC2 expression in INs of Dlx5 KCC2 cKO. **(A)** KCC2 expression in WT (top row) and Dlx5 KCC2 cKO (bottom row) cortical hemisections at P0, P13, and P19. Note lack of KCC2 immunoreactivity in somatosensory cortex (SSC) of Dlx5 KCC2 cKO at P0, but not at later ages. Loss of immunoreactivity persists in striatum (Str), hippocampal hilus (hyl), and reticular nucleus of thalamus (nRT). Regions of interest are outlined by a dashed white line. **(B)** Close-up images showing loss of plasmalemmal KCC2 immunoreactivity in INs marked by cre tdTomato reporter (KCC2 green, INs red). Arrows point out several examples of KCC2-expressing INs in WT.

This finding is in line with previous work suggesting that Cl^–^ transporters regulate even the earliest stages of IN development, including migration. We hypothesized that precocious KCC2 expression is important for development and emerging circuit function of INs. We created a conditional knockout mouse (Dlx5 KCC2 cKO) that lacks expression of KCC2 in Dlx5-lineage neurons. This was done by breeding Dlx5:cre-IRES-EGFP line with a line that has *lox*P sites surrounding exon 5 of *Slc12a5*. Expression of cre in a cell typically results in full loss of KCC2 expression (Mavrovic et al., 2020). Mice were also bred to include a cre-driven Ai14 tdTomato reporter to track Dlx5-expressing INs. In Dlx5 KCC2 cKO cortex, KCC2 immunoreactivity is completely absent at P0 (Figure 2), confirming that KCC2 expression in neonatal cortex is exclusive to INs. At P13 and P19, expression of KCC2 is comparable in wild-type and Dlx5 KCC2 cKO cortices, which attests to unaffected KCC2 expression in principal neurons. However, brain structures that are predominantly populated by Dlx5-lineage neurons have a profound loss of KCC2 expression at all timepoints. This includes medium spiny neurons of the striatum, neurons of reticular nucleus of the thalamus (Figure 2A and Supplementary Figure 1), and granule cells of the olfactory bulb (Supplementary Figure 2).

### Changes in Interneuron Distribution in Dlx5 KCC2 cKO Mouse

Multiple studies demonstrate that GABA provides the impetus for migration of INs in the marginal zone (Cuzon et al., 2006; Inada et al., 2011), and that the onset of KCC2 expression coincides with the timepoint when INs reach their final intracortical locations and stop migrating (Bortone and Polleux, 2009; Inada et al., 2011; Inamura et al., 2012). Therefore, we hypothesized that INs in Dlx5 KCC2 cKO mice would remain in a prolonged migratory state, producing an altered cortical distribution of INs. Migrating INs first enter the cortex laterally and then migrate in the marginal zone and intermediate zone toward more dorsomedial areas. Upon entering the cortical plate, they first populate deep cortical layers before superficial layers. Prolonged migration could then shift IN distribution to enrich areas that form later in development, such as superficial cortical layers and medial cortex.

**FIGURE 3:**
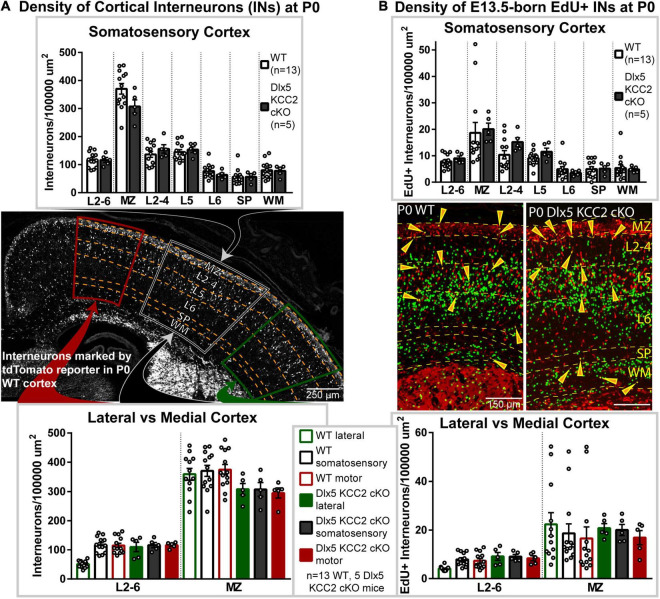
Interneuronal loss of KCC2 does not alter perinatal IN migration. **(A)** Distribution of INs, marked by tdTomato cre reporter, in P0 Dlx5 KCC2 cKO and age-matched WT. Top graph compares cortical layers of somatosensory cortex, and bottom compares medial and lateral cortex. Center image shows exemplar distribution of INs in P0 WT cortex with labels of layers/regions used for comparison. **(B)** Distribution of E13.5-labeled EdU+ INs in P0 Dlx5 KCC2 cKO and age-matched WT. EdU was injected into G13.5 pregnant dams, and distribution of EdU+ INs (shown in center images) was compared between Dlx5 KCC2 cKO and WT in cortical layers of somatosensory cortex (top graph), and between lateral and medial cortex (bottom graph). For both **(A,B)**, no significant differences found by one-way ANOVA with Sidak’s multiple comparisons. Comparisons were also made between individual layers of medial and lateral cortex with no differences found (data not shown). For **(A,B)**, *n* = 13 WT and 5 Dlx5 KCC2 cKO mice. Abbreviations: (MZ), marginal zone; (L2-4), layers 2-4; (L5), layer 5; (L6), layer 6; (layers 2-6), L2-6; (SP), subplate; (WM), white matter.

To evaluate if this shift occurs in Dlx5 KCC2 cKO, we quantified IN distribution during the final stages of cortical migration at P0. Surprisingly, we did not find an enrichment of INs in the migratory marginal zone, nor did we observe a shift in IN distribution among cortical layers or between the medial and lateral regions of Dlx5 KCC2 cKO cortex (Figure 3A).

### Normal Distribution of Birth-Dated Interneurons

Normal cortical development involves several different cell types, each of which may develop at different rates. While IN distribution at P0 appeared normal, we hypothesized that loss of KCC2 may disrupt the tempo of IN migration, causing INs to end migration out of phase with other circuit-forming processes in perinatal cortex. Therefore, we used a birth dating procedure, labeling newborn INs with EdU at E13.5 and determining their location at P0. Normally, some E13.5-born INs are still migrating at P0, while others have entered the cortical plate (Figure 3B), allowing us to test for a delayed exit from the migratory zones and for shifts in IN distribution. Somewhat surprisingly, we found no difference in the density of migrating E13.5-born INs between wild-type and KO mice (Figure 3B). Contrary to our hypothesis, INs in Dlx5 KCC2 cKO were distributed similarly to wild-type, both when comparing regions along the mediolateral axis and across cortical layers (Figure 3). For both WT and Dlx5 KCC2 cKO, there were more EdU+ INs in deeper layers of medial cortex than lateral cortex, in line with lateral-to-medial sequence of development for both principal neurons and INs (Smart, 1984), though these medial-to-lateral differences were much more subtle for INs than for all other EdU+ cells. These results indicate that despite loss of KCC2, INs could exit migratory streams in a timely manner and navigate to their typical positions.

### Dlx5 KCC2 cKO Have Failure to Thrive, Seizures and Early Death

**FIGURE 4:**
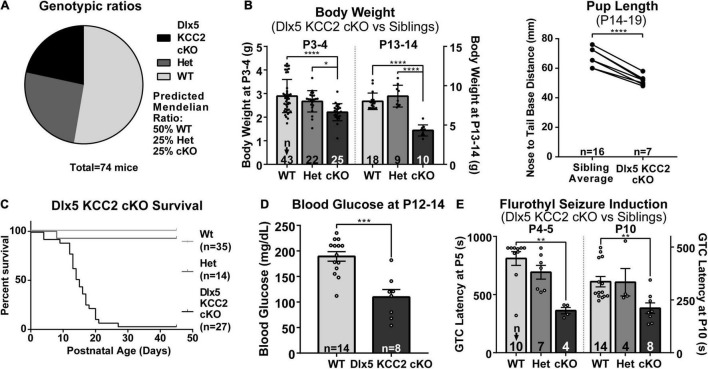
Dlx5 KCC2 cKO mice exhibit failure to thrive and seizures. **(A–C)** Graphs showing that Dlx5 KCC2 cKO pups are born at expected 25% Mendelian ratio **(A)**, but have decreased weight and length **(B)**, and die in the postnatal period **(C)**. One-way ANOVA with Sidak’s multiple comparisons: *P < 0.05, ****P < 0.0001. For body length, paired comparisons against an average of matched littermate lengths were done using a ratio paired T-test, ****P < 0.0001. **(D)** Mild hypoglycemia in Dlx5 KCC2 cKO pups. T-test: ***P < 0.001. **(E)** Susceptibility of Dlx5 KCC2 cKO pups to faster onset of flurothyl-induced generalized tonic clonic seizures (GTC) at P4–P5 and P10. Kruskal–Wallis non-parametric ANOVA: **P < 0.01. The number of mice examined is indicated by n values in each panel. For bar graphs, these are listed at the bottom of each bar.

Despite appearing relatively normal at birth, the Dlx5 KCC2 cKO mice exhibited a severe failure to thrive phenotype at later ages. While Dlx5 KCC2 cKO pups are born at expected Mendelian ratios, and no pups died prenatally, Dlx5 KCC2 cKO pups had reduced body weight and body length than their littermates, were mildly hypoglycemic, had occasional spontaneous seizures, and died in the third to fourth postnatal weeks (Figures 4A–D). Previous work has sometimes found that genetic disruption of GABAergic signaling can produce unexpected anatomic defects, such as the cleft palate found in *Gabrb3* knockout mice (Culiat et al., 1995). Both Dlx5 and KCC2 have relatively limited areas of tissue expression, and the combination of these two makes it unlikely that our genetic model has severely disrupted KCC2 expression outside the nervous system. Nonetheless, we performed necropsy and found no obvious anatomical or histological defects in any of the major organ systems. The palatal structures form normally in Dlx5 KCC2 cKO, and Dlx5 KCC2 cKO pups can be observed feeding and display a “milk spot” of consumed milk in their stomach. Thus, we believe Dlx5 KCC2 cKO pups have no physical impediment to feeding. Therefore, we expect that failure to thrive and early death is due to a developmental or neurological condition. We did not find any gross differences in brain morphology of P17 Dlx5 KCC2 cKO mice, and vGlut2 (Supplementary Figure 3) immunofluorescence showed a grossly normal laminar pattern of somatosensory cortex.

### Spontaneous Seizures and Induced-Seizure Susceptibility in Dlx5 KCC2 cKO Mouse

There is extensive literature on KCC2 dysfunction with epilepsy, and our mice had occasional epileptic seizures. Therefore, we decided to more formally evaluate seizure susceptibility of Dlx5 KCC2 cKO by inducing generalized tonic-clonic seizures in P4–P5 and P10 pups with flurothyl. These ages were chosen based on the fact that KCC2 expression is relatively low in non-INs at P5 and earlier, but more diffusely expressed after P10 (Takayama and Inoue, 2010). We found that from an early postnatal age, Dlx5 KCC2 cKO pups developed seizures significantly faster than their wild-type and heterozygous littermates (Figure 4E). Moreover, video monitoring revealed that Dlx5 KCC2 cKO pups frequently wandered out of the nest and exhibited spontaneous seizures (Supplementary Movie 1) much like the global KCC2b knockout mice (Woo et al., 2002). Finally, we used overnight video monitoring to help clarify the cause for early death in the Dlx5 KCC2 cKO mice. These recordings were done in the animal’s home cage with their mothers and littermates. Therefore, we could not visualize the entire cage all night. Nonetheless, in 6 out of 9 monitored cases with early death, Dlx5 KCC2 cKO pups experienced a violent and prolonged seizure the night before their death (Supplementary Movie 1). These findings lead us to believe that loss of KCC2 in Dlx5 lineage causes mice to have seizures that disturb normal pup behavior and eventually lead to their death.

### Increased Interneuron Density in Layer 5 at P12–14, but Unchanged Somatic Inhibition in Pyramidal Cells

**FIGURE 5:**
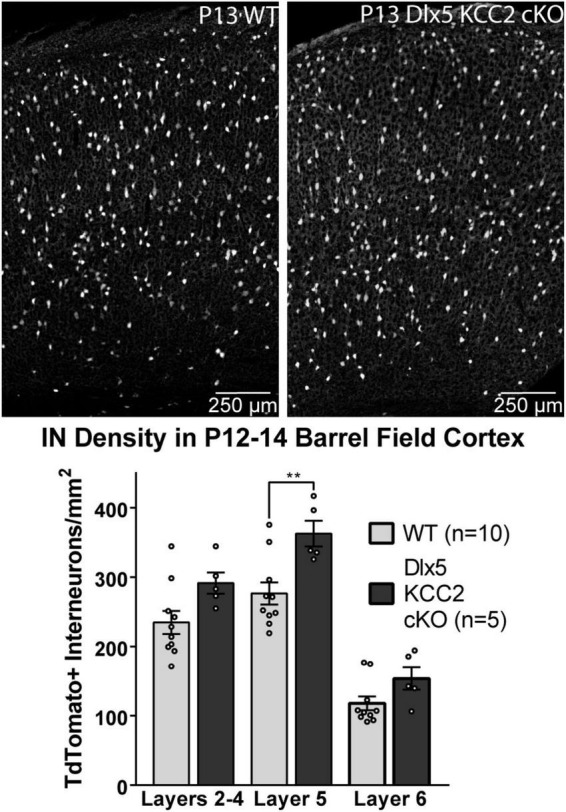
Increase in Layer 5 INs in P12–14 Dlx5 KCC2 cKO Cortex. Bar graph shows densities of INs, labeled by cre reporter, by different layers of barrel field cortex in P12–14 WT, heterozygote, and Dlx5 KCC2 cKO. Images show examples of IN reporter expression in WT and Dlx5 KCC2 cKO cortex. One-way ANOVA with Sidak’s multiple comparisons: **P < 0.01. *n* = 10 WT, 5 Dlx5 KCC2 cKO mice.

The seizure phenotype of Dlx5 KCC2 cKO suggested a deficiency in IN function in Dlx5 KCC2 cKO pups, a potential origin of which is altered IN distribution. Despite a normal distribution of INs at P0, a shift could occur later due to altered cell-type specific survival. Therefore, we evaluated IN distribution in P12–14 barrel cortex, as early death precluded rigorous examination of later ages. At P12–14, we detected a ∼30% increase in density of layer 5 INs (Figure 5).

**FIGURE 6:**
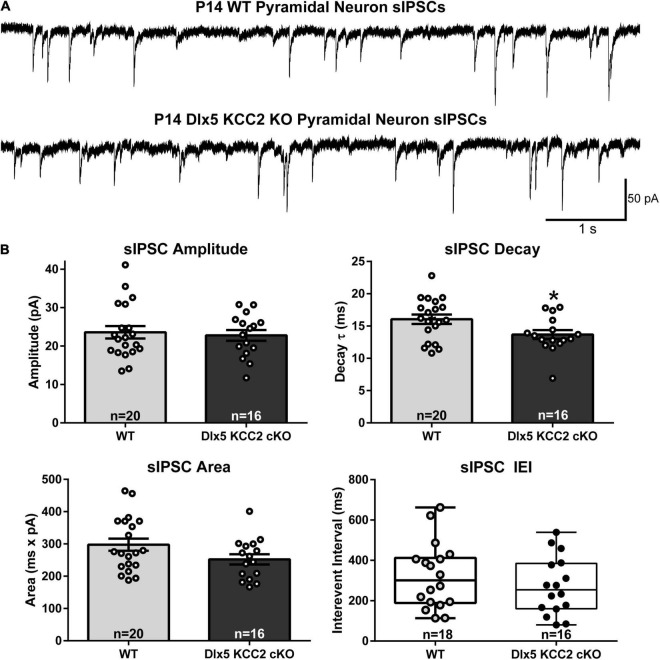
Normal sIPSC frequency, faster decay in pyramidal neurons in KO. **(A)** Sample recordings of spontaneous IPSCs in layer 5 pyramidal neurons in barrel cortex of P12–14 WT and Dlx5 KCC2 cKO. **(B)** Amplitude, area, decay (mean ± SEM), and inter-event interval (IEI, box and whisker plot showing median, 25/75 percentile, and maximum/minimum) of spontaneous IPSCs in layer 5 pyramidal neurons in barrel cortex of P12–14 WT and Dlx5 KCC2 cKO. *T*-test: *P < 0.05. Non-parametric Mann–Whitney test was used for IEI only. *n* values at the bottom of each bar/box and whisker plot indicate the number of cells recorded, taken from ≥5 mice, for each group.

We therefore hypothesized that excess IN density in layer 5 would cause disrupted inhibition in that layer. Contrary to this hypothesis, we recorded normal frequency of sIPSCs from layer 5 pyramidal neurons (Figure 6), as well as normal amplitude and total charge transfer, and a slightly faster decay than WT.

### Changes in Parvalbumin and Somatostatin Subtypes

**FIGURE 7:**
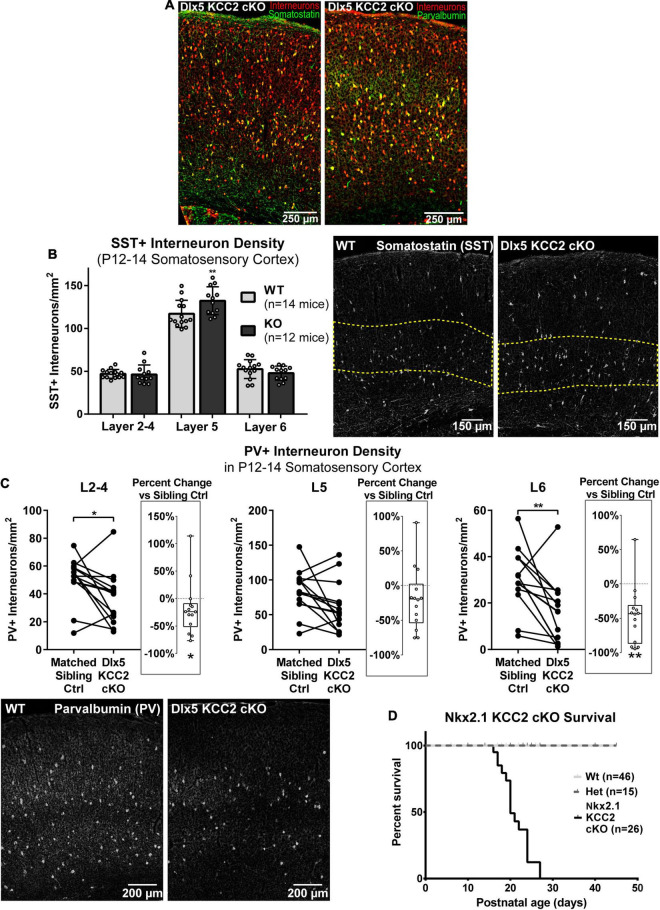
Altered distribution of somatostatin and parvalbumin INs in maturing cortex of Dlx5 KCC2 cKO. **(A)** Images showing complete co-localization of SST (left, green) and PV (right, green) immunopositive neurons with Ai14 cre reporter used to label INs (red) in P13 barrel field cortex. **(B)** Average density with SEM of SST+ cells in different layers of P12–14 somatosensory cortex with exemplary images of SST immunolabeling. Yellow dashed line demarcates layer 5. *n* = 14 WT, 12 Dlx5 KCC2 cKO mice. One-way ANOVA with Sidak’s multiple comparisons: **P < 0.01. **(C)** Paired comparisons of density of PV+ cells in layers 2–4 (L2–4), 5 (L5), and 6 (L6) in P12–14 somatosensory cortex of Dlx5 KCC2 cKO and control littermates, with exemplary images of PV immunolabeling. Box and whisker plots in each graph plot percent difference change in each littermate pair, *n* = 12 littermate mouse pairs. Wilcoxon matched-pairs signed rank test, **P < 0.01, *P < 0.05. **(D)** Survival plot of Nkx2.1 KCC2 knockout, conditional only to PV and SST INs. *n* = 46 WT, 15 heterozygote, 26 Nkx2.1 KCC2 knockout mice.

By recording from layer 5 pyramidal somata, our slice physiology experiments were biased toward measuring disruption in intralaminar parvalbumin (PV)-expressing basket cell input. One reason for unaltered sIPSCs in layer 5 may be because Dlx5 KCC2 cKO affected non-PV cells. The majority of layer 5 INs express either PV or somatostatin (SST), so we evaluated whether either of these populations is selectively affected in Dlx5 KCC2 cKO. Per expectation, SST and PV immunostaining labeled non-overlapping populations, fully co-localized with our IN reporter, and each labeled about 30% of tdTomato+ INs (Figure 7A). Because parvalbumin expression begins at mature developmental stages, numbers of PV+ INs significantly varied between P12–14 animals due to slight differences in their age and development. Therefore, we made paired comparisons of PV+ IN densities between siblings, but a grouped comparison of SST+ INs. We found that at P12–14, SST+ INs showed a modest, but significant density increase in L5 and were comparable to wild-type siblings in other layers of somatosensory cortex (Figure 7B), although this finding was not preserved in the small subset of Dlx5 KCC2 cKO that survived until P18–20 (Supplementary Figure 4). On the other hand, there was a significant decrease in density of PV+ INs in layers 2–4, layer 6, but not layer 5 in somatosensory cortex at P12–14 (Figure 7C). We therefore concluded that Dlx5 KCC2 cKO mice suffer a layer-specific PV and SST IN disruption.

However, whether the change in PV and SST INs significantly contributes to Dlx5 KCC2 cKO pathology was unclear due to potential confounding effects from other GABAergic cells affected by KCC2 loss. Thus, we next investigated whether loss of KCC2 in a more restricted population of INs would result in a similar phenotype to Dlx5 KCC2 cKO using Nkx2.1 driven cre expression to inactivate KCC2 in medial ganglionic eminence (MGE) precursors (Nkx2.1 KCC2 cKO). Nkx2.1-cre is minimally active in caudal ganglionic eminence, thus targeting fewer IN subtypes than Dlx5, being primarily limited to SST and PV INs within cortex (Xu et al., 2008). Nkx2.1 KCC2 cKO pups also died at or before weaning, indicating that relatively selective loss of KCC2 in PV and SST INs can bring about a pathology similar to Dlx5 KCC2 cKO (Figure 7D).

### Normal Composition of GABAergic Synapses

We also wanted to assess for changes in the number and composition of inhibitory synapses in our Dlx5 KCC2 cKO mice. Depolarizing GABA is reported to guide synaptogenesis, including that of GABAergic synapses. The number or composition of GABAergic synapses could thus be altered in Dlx5 KCC2 cKO, which could underlie the seizure phenotype. For this reason, we looked for changes in density of GABAergic synapses by counting vGAT puncta, which mark presynaptic terminals, and found comparable densities in each layer of somatosensory cortex in WT and Dlx5 KCC2 cKO (Supplementary Figure 5). Furthermore, we found no gross differences in expression of GABA_A_ receptor subunits (Supplementary Figure 6), which undergoes a dramatic shift during development and imparts significant changes to synaptic signals.

### Parvalbumin KCC2 cKO Mouse Has Milder Phenotype Than Dlx5 KCC2 cKO Mouse

**FIGURE 8:**
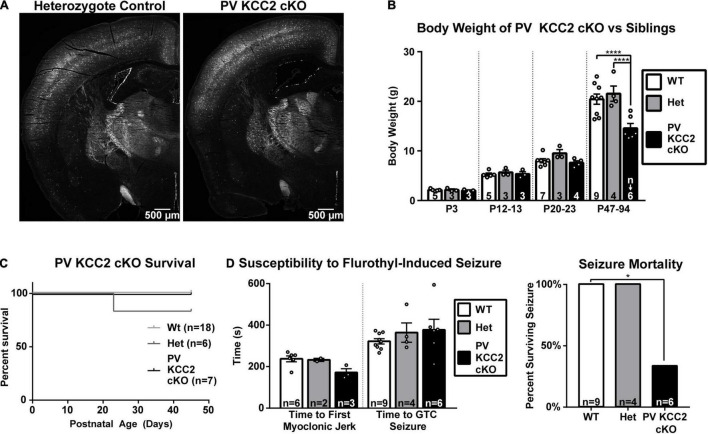
Selective loss of KCC2 in parvalbumin neurons does not cause seizure susceptibility nor failure to thrive. **(A)** PV INs labeled by Ai14 cre reporter in hemisections of P64 PV KCC2 cKO and sibling control. **(B)** Body weight of PV KCC2 cKO. One-way ANOVA with Sidak’s multiple comparisons: ****P < 0.0001. Note normal PV KCC2 cKO body weight until adulthood, when it is slightly diminished. **(C)** Postnatal survival is unaffected in PV KCC2 cKO. **(D)** Seizure susceptibility (right) measured by time to first myoclonic jerk and generalized tonic clonic seizure (left) and seizure mortality (right) resulting from flurothyl seizure induction in PV KCC2 cKO mice. Note that PV KCC2 cKO do not exhibit faster onset of induced seizures, but are highly likely to die from them. Chi squared with Yates’ correction: *P < 0.05. The number of mice examined is indicated by *n* values in each panel. For bar graphs, these are listed at the bottom of each bar.

We hypothesized that a significant portion of Dlx5 KCC2 cKO phenotype arises from loss of KCC2 in PV INs. These INs provide somatic inhibition to local pyramidal neurons and serve as primary mediators of local feedforward and feedback inhibition, such as in the thalamocortical circuit. A change in the number or excitability of these cells would hinder feedforward and feedback inhibitory regulation of the cortical circuit, and could lead to seizures that we observe in Dlx5 KCC2 cKO. We observed a decrease in cortical PV+ cell density in the Dlx5 KCC2 cKO mouse. Moreover, a significant decrease of PV+ cells is reported in hippocampus of global KCC2b knockout (Woo et al., 2002). To distinguish the contribution of PV-driven pathology to Dlx5 KCC2 cKO mouse phenotype, we created a conditional KCC2 knockout using the parvalbumin promoter, which restricts KCC2 loss to maturing PV interneurons starting from the second postnatal week, but leaves perinatal immature PV neurons and other INs types unaffected. Unlike Dlx5 KCC2 cKO mice, the PV KCC2 cKO mice had normal pup weight and survived to adulthood, though adult weight of PV KCC2 cKO was slightly lower than wildtype littermates (Figures 8B,C). Moreover, the density of PV INs, labeled by the cre reporter, appeared comparable to heterozygote control (Figure 8A). PV KCC2 cKO mice develop flurothyl-induced seizures with the same latency as wild-type littermates (Figure 8D), but have a high likelihood of fatality, as 4 out of the 6 tested PV KCC2 cKO mice died shortly following the seizure. PV KCC2 cKO mice also developed an adult-onset motor phenotype, marked by reduction of muscle tone and tremor when moving limbs (Supplementary Movie 2). The phenotype of Dlx5 KCC2 cKO mice thus arises from early and pan-IN loss of KCC2, and is not driven solely by PV INs.

## Discussion

KCC2 is an important regulator of neuronal development, particularly for INs, where timely expression of KCC2 has been hypothesized to stop IN migration. We demonstrated that cortical expression of KCC2 in INs begins as early as in the embryonic period, which is well-timed to terminate IN migration and subsequent early circuit formation. To investigate the role of KCC2 in development of INs, we created the conditional Dlx5 KCC2 cKO mouse that lacks KCC2 in forebrain GABAergic neurons. We showed that despite loss of KCC2 expression, INs migrated to their normal cortical destinations in the perinatal period without obvious delay. Nonetheless, the Dlx5 KCC2 cKO mice have failure to thrive and spontaneous seizures leading to early death around the third week of life. Potentially, this pathology is related to layer and subtype-specific imbalance of IN distribution, though subcortical effects of the Dlx5 KCC2 cKO, which were not the focus of this investigation, might also be a contributing factor.

KCC2 is clearly highly important to IN function. The seizure pathology and failure to thrive of Dlx5 KCC2 cKO mice is very similar to a global KCC2b knockout mouse, suggesting that much of the previously mentioned KCC2b global knockout pathology is an interneuronopathy. Though we observed a mild hypoglycemia in Dlx5 KCC2 cKO, blood glucose levels of Dlx5 KCC2 cKO remained higher than those reported to cause seizures (Schauwecker, 2012). This is in line with observations in KCC2b global knockout, which has a malnutrition phenotype, but rescuing this phenotype with supplemental feeding does not prevent seizures nor death, though it does temporarily rescue weight loss. Therefore, we believe seizures in both mice originate from malfunctioning INs.

At the outset of this study, we hypothesized the transporter function of KCC2 to be a regulator of IN development. Early GABA signaling promotes many aspects of neuronal development, and changes in that system can lead to abnormal circuit formation. Developmental processes like neurite arborization (Chattopadhyaya et al., 2007) and IN migration (Cuzon et al., 2006; Inada et al., 2011) are excessive if GABA levels or signaling are increased. Conversely, migration is stalled and morphological development is stunted when GABA is diminished by drugs, reduction in GABA synthesis (Cuzon et al., 2006; Inada et al., 2011), or made prematurely hyperpolarizing by KCC2 expression (Cancedda et al., 2007; Bortone and Polleux, 2009). Moreover, intrinsic mechanisms regulating transporter activity, such as KCC2 expression and post-translational modifications (Fukuda and Watanabe, 2019), allow neurons to interpret a similar developmental environment in a manner appropriate to each neuron’s stage of maturity. For instance, individual maturing INs leave a migratory zone while other INs, surrounded by the same cues, continue migrating. For these reasons, the onset of endogenous KCC2 expression was thought to carry out an important regulatory role by defining a cell-autonomous temporal window for developmental processes, especially termination of IN migration. Contrary to this hypothesis, our experiments demonstrate that IN migration occurs normally when KCC2 is deleted. We conclude that the onset of KCC2 expression is not the sole determinant of the exit from migration, and other compensatory mechanisms must be in place to transition INs to a mature, post-migratory state. However, our observations are limited by the timepoints that we chose for our experiments. It is possible that KCC2 loss causes transient migratory defects or movement speed, but not location of migratory INs on a population level. These changes would best be detected by more thorough means: time-lapse imaging of migratory INs or measurements of cortical IN distribution throughout many timepoints.

It is striking that deleterious effects of KCC2 loss impact Dlx5 KCC2 cKO mice at an early age, with small weight and seizure susceptibility detectable as early as P4–P5, when KCC2 expression in cortex is low (Takayama and Inoue, 2010) and generally restricted to INs. At this age, *in vitro* studies report that GABA is depolarizing to pyramidal neurons (Rheims et al., 2008), and that giant depolarizing potentials (GDPs) that are driven by GABA help incorporate deep layer neurons into functional circuits (Allene et al., 2008; Griguoli and Cherubini, 2017). One might speculate that seizure susceptibility at P4–P5 could indicate increased IN activity and increased GDP output, as well as globally increased activity. However, by P10–P14, pyramidal neurons express KCC2, and GABA-driven GDPs have largely disappeared. The fact that we still see seizure susceptibility at this age suggests that IN-specific knockout may cause a change in network formation that persists even after GABA becomes mostly inhibitory in the brain. At the same time, we cannot rule out the possibility that hyperexcitable interneurons, independent of any developmental effects, are the source of seizures and early death in our Dlx5 KCC2 cKO mice. This might be answered using an inducible, interneuron-specific promoter to knockout KCC2 later in life. However, this is beyond the scope of the current paper.

While our initial hypotheses were based on the literature showing that KCC2 and depolarizing GABA regulate IN development, it is important to note that the KCC2 function is not limited to Cl^–^ transport. A rapidly growing body of literature suggests that KCC2 may also regulate neuronal development and function through a number of protein-protein interactions. The most well-studied is regulation of dendritic spine formation and long-term synaptic plasticity through direct protein/protein interactions with β-pix and/or the cytoskeletal protein 4.1N (Li et al., 2007; Llano et al., 2015, 2020), and regulation of AMPA receptor trafficking through transport-independent cofilin phosphorylation (Gauvain et al., 2011; Chevy et al., 2015). Furthermore, KCC2 expression is associated with apoptosis. Premature overexpression of KCC2 disrupted brain development through a transport-independent interaction with the cytoskeleton-associated protein 4.1N (Horn et al., 2010), while loss of KCC2 leads to excessive perinatal apoptosis (Mavrovic et al., 2020), and KCC2 overexpression and pharmacological block lead to apoptosis in mature neurons (Kontou et al., 2021). Studies of protein/protein interactions and work using transporter-deficient KCC2 mutants have revealed a previously unsuspected array of activity through interactions through membrane and cytoskeletal proteins (Llano et al., 2020), such as kainate receptors (Mahadevan et al., 2014), β-Pix (Llano et al., 2015), KCNK9 channels (Goutierre et al., 2019), gephyrin (Al Awabdh et al., 2021), GABA_B_ receptors (Wright et al., 2017), and Neto2 (Ivakine et al., 2013). Some of these interactions have effects independent of GABAergic signaling, such as increasing neuronal excitability through downregulating KCNK9 expression (Goutierre et al., 2019). Other interactions currently appear to be mechanisms for regulating transporter activity of KCC2 (Ivakine et al., 2013; Mahadevan et al., 2014), but likely have additional effects. This field is still in its infancy, and much of this work has targeted exclusively glutamatergic neurons. Therefore, the extent to which these interactions regulate IN development and function is yet to be determined.

A question still remains of whether synapses or neurite arbors of INs developed differently in Dlx5 KCC2 cKO. INs do not form significant dendritic spines and are less likely to rely on some of the scaffolding roles of KCC2. Therefore, we did not investigate glutamatergic synapses of INs. However, KCC2 may still be involved in formation and plasticity of glutamatergic synapses in INs, which a follow-up study might investigate. On the other hand, GABA has been shown to promote neurite arborization and synaptogenesis of GABAergic synapses in multiple neuronal types (Gascon et al., 2006; Ge et al., 2006; Bouzigues et al., 2007; Cancedda et al., 2007; Chattopadhyaya et al., 2007; Oh et al., 2016; Brady et al., 2018) and recently, an interaction between KCC2 and GABAergic synapse scaffolding protein gephyrin has been demonstrated (Al Awabdh et al., 2021). Nonetheless, we found unremarkable expression of synaptic proteins and sIPSCs in layer 5 pyramidal neurons, suggesting normal development of GABAergic synapses in Dlx5 KCC2 cKO cortex. Possibly, KCC2 does not affect synaptic development of INs, despite the circumstances that suggest involvement. For example, synaptogenesis of glutamatergic and GABAergic synapses within the lateral superior olive is not affected by loss of KCC2, despite coincident onset of KCC2 expression and critical window of synapse formation (Lee et al., 2016). However, it may also be true that more subtle changes in synapse formation occur, since our analysis can only detect gross changes in GABAergic synapses. To thoroughly investigate effects of KCC2 loss on IN synaptogenesis/neurite formation, a follow-up study is needed, focusing on the morphology of neuronal arbors as well as the density, localization, and composition of inhibitory synapses. Moreover, since INs are morphologically diverse, the study must selectively analyze IN subtypes and cortical layer locations.

While IN migration was unaffected, loss of KCC2 increased IN density in layer 5 at 2 weeks of age. Considering normal IN migration and perinatal IN counts, this was presumably due to increased cell survival. If this effect is mediated by a prolonged period depolarizing GABA, it contrasts with previous studies. Only exceptionally strong alterations in GABAergic excitation were shown to affect cell survival (Represa and Ben-Ari, 2005), primarily causing decreased neuron numbers. For example, application of a high concentration of GABAergic agonist muscimol caused death of GABAergic neurons in 3D cultures (Honegger et al., 1998); in contrast, embryonic application of GABAergic antagonist also caused loss of PV INs (Luk and Sadikot, 2001). Increased survival could be mediated by increased expression of neurotrophins (Ceni et al., 2014), such as BDNF, in response to GABAergic depolarization (Obrietan et al., 2002; Fukuchi et al., 2014; Brady et al., 2018). However, while limited studies show that neurotrophins are produced by INs (Biane et al., 2014; Barreda Tomás et al., 2020) and regulate certain aspects of IN development/plasticity (Jones et al., 1994; Polleux et al., 2002; Jin et al., 2003; Patz et al., 2004), their role in IN survival is not known. Independently of transport function of KCC2, both overexpression and loss of KCC2 have also been reported to promote apoptosis during development (Horn et al., 2010; Mavrovic et al., 2020). Somewhat more consistent with our findings, recent work in hippocampal pyramidal cells showed that KCC2 loss promotes apoptosis after 18 days *in vitro*, but not at 3 days, which is a more relevant timeframe for most of our work (Kontou et al., 2021). Interestingly, despite an increase in layer 5 INs, we did not observe a change in inhibitory synaptic input received by pyramidal neurons. It is possible that while IN numbers changed, the number of synapses remained constant through compensation. Alternatively, our recordings were done at room temperature and may have missed changes seen at more physiologically relevant temperatures, though there is no *a priori* reason to think this is the case. Recordings from WT and KO tissue were done at the same temperatures, and the scant literature regarding temperature dependence of KCC2 shows that it is likely insensitive (Woodin et al., 2003) or suppressed at higher temperatures (Hartmann and Nothwang, 2011). An equally plausible explanation is that whole cell recordings reflect perisomatic input, which is chiefly provided by PV INs, which were present at normal density in layer 5 of Dlx5 KCC2 cKO. Therefore, while SST INs were increased in layer 5, they may have not influenced whole cell recordings due to their typical projection to distal dendrites.

Additionally, outside of layer 5, we found that loss of KCC2 caused a pronounced loss of PV+ INs, similar to what has been reported in the global KCC2b knockout (Woo et al., 2002). Impaired development or dysfunction of PV INs have also been associated with epilepsy (Jiang et al., 2016). PV INs regulate cortical excitation and oscillatory activity, and in pathologic conditions, seizure initiation and spreading often depends on PV IN activity (Jiang et al., 2016; Anstotz et al., 2021), where PV INs can either promote seizures by synchronizing circuit firing or prevent seizure spreading through inhibition. Given the loss of PV+ INs both in Dlx5 KCC2 cKO and global KCC2b knockout, we hypothesize PV IN dysfunction may play an important role in the observed phenotypes. However, it is unclear whether the loss of PV+ INs reflects neuronal death, thereby decreasing cortical inhibition, or simply lost parvalbumin protein expression. Though the mechanism is not known, reduced parvalbumin expression is associated with a number of psychiatric disorders (ASD, schizophrenia) (Filice et al., 2020; Ruden et al., 2021) and can cause increased network synchronization/enhanced PV IN output by enhancing facilitation and shortening delayed transmitter release in INs that are deficient in PV expression (Collin et al., 2005; Muller et al., 2007; Manseau et al., 2010; Orduz et al., 2013).

It is possible the seizures and early death in our mice arise from pan-interneuronal dysfunction due to the loss of KCC2. We tested whether cell-restricted loss of KCC2 in mature PV INs was sufficient to cause a significant pathology similar to IN-wide KCC2 loss by using a mouse with PV-specific conditional KCC2 knockout. Since PV gene expression in cortex is absent before the second postnatal week (Alcántara et al., 1993; del Rio et al., 1994), the PV KCC2 cKO animals have normal KCC2 expression during development, but loss of KCC2 in mature PV neurons. The PV KCC2 cKO mouse did not have the seizure susceptibility, failure to thrive pathology, nor prominent PV IN loss. Therefore, the seizures and premature death we observe in Dlx5 KCC2 cKO after P10 does not solely arise from hyperexcitability in mature PV INs. This could suggest that PV neurons do not play a role in the phenotype of our Dlx5 KCC2 cKO mice. However, another possibility is that the effects of KCC2-knockout are specific to the perinatal period. Interneuron dysfunction in the early postnatal period may produce developmentally-specific derangement of circuit formation and function through a variety of mechanisms, including early network oscillation/GDP synchronization and activity-dependent growth factor release (Jin et al., 2003; Patz et al., 2004; Biane et al., 2014; Brady et al., 2018). This interpretation is also supported by seizure susceptibility of Dlx5 KCC2 cKO mice at P4–5. Development of IN subtypes is tightly interdependent through complex and nuanced mechanisms (Duan et al., 2020). For instance, SST INs play a vital role in orchestrating the establishment of PV-neuron feed-forward inhibition in perinatal thalamocortical circuits (Tuncdemir et al., 2016). Moreover, MGE-derived interneurons form spatially segregated neuronal assemblies with excitatory neurons, before large scale circuits become established. Loss of this GABAergic input disrupts normal apoptosis, leading to excess survival of SST and PV INs (Duan et al., 2020). In Dlx5 KCC2 cKO, changes in inhibition and synchrony due to hyperexcitability of INs in Dlx5 KCC2 cKO could likewise lead to changes in SST and PV IN densities.

To conclude, we found that KCC2 is vital for IN development and function, but despite models in the field, is not the primary cue for terminating IN migration. Deficits in IN distribution specific to cortical layers and IN subtype-specific deficits in IN distribution likely underlie the seizures suffered by Dlx5 KCC2 cKO, but further studies targeting physiology and structure of INs, as well as temporally restricting KCC2 loss, would uncover the full breadth of developmental and physiological deficits contributing to this phenotype.

## Materials and Methods

### Animal Husbandry

The Tg(mI56i-cre-EGFP)1Kc (Dlx5/6-Cre-IRES-EGFP) (Stenman et al., 2003), Tg(Nkx2-1-cre)2S and (Nkx2.1-cre), Pvalb^TM 1(*cre*)*Arbr*^ (PV-cre), and Gt(ROSA)26Sor^TM 14(CAG–*tdTomato*)*Hze*^ (Ai14) (Madisen et al., 2010) mice were acquired from Jackson laboratories (Stock #007914, 008661, 008069, and 023724, respectively). The KCC2 flox mouse was created and contributed by Dr. Eric Delpire. In this mouse, *lox*P sites flank exon 5 of *Slc12a5*, and expression of cre in a cell results in excision of exon 5, premature stop codon, and complete KCC2 knockout (Mavrovic et al., 2020). PV-cre, Nkx2.1-cre, and Ai14 were maintained in C57/Bl6 background, while Dlx5/6-cre-IRES-EGFP and KCC2 flox were in FVB background, resulting in mixed B6/FVB mice for majority of Dlx5 KCC2 cKO experiments.

### Weight and Length Measurement

Body weight was measured by placing a pup into a plastic beaker or large weight boat on a scale. Body length was measured by anesthetizing pups with isoflurane, and suspending them vertically by the tail. Nose tip to tail base length was measured against a ruler. Pups that were anesthetized for body length measurement were not used in subsequent studies.

### Genotyping, PCR and Primers

Genomic DNA extraction and PCR were performed on mouse biopsy tissue (tail or toe if P0–P7, ear lobe if P12+) using Sigma REDExtract-N-Amp Tissue PCR kit Catalog#: XNAT-100RXN (Sigma, St. Louis, MO, United States). Target bands were distinguished by electrophoresis on a 2% agarose gel stained with SYBR Safe DNA gel stain from Invitrogen Cat# P/N S33102 and visualized using Bio-Rad Gel Doc EZ.

For genotyping our Ai14-tdTomato mice, we used the following primers: WT forward 5′-AAGGGAGCTGCAGT GGAGTA-3′; WT reverse 5′-CCGAAAATCTGTGGGAAGTC-3′; Ai14 forward 5′-CTGTTCCTGTACGGCATGG-3′; Ai14 reverse 5′-GGCATTAAAGCAGCGTATCC-3′. The reaction conditions were 94°C for 5 min, (94°C for 15 s, 65°C for 1 min, 72°C for 30 s) × 36 cycles, 72°C for 2 min. Product bands were 297 bp WT, 196 bp Ai14 positive.

For genotyping our floxed KCC2 mice, we used the following primers: forward 5′-TTACACAAGTACTGCCGGTCCATTG-3′; reverse 5′-GCCTCAAGGCTATGTGTAAAGACTCA-3′. The reaction conditions were 94°C for 5 min, (92°C for 30 s, 62°C for 30 s, 72°C for 30 s) × 40 cycles. Product bands were 230 bp WT, 282 bp KCC2 floxed.

For genotyping our Dlx5:Cre-IRES-EGFP and PV-cre mice, we used the following primers targeting the Cre sequence forward 5′-GCATTACCGGTCGATGCAACGAGTGATGAG-3′; reverse 5′-GAGTGAACGAACCTGGTCGAAATCAGTGCG-3′. The reaction conditions were 94°C for 3 min, (94°C for 30 s, 68°C for 30 s, 72°C for 1 min) × 36 cycles. Product band was 408 bp.

### Blood Glucose Measurement

Blood glucose was measured with OneTouch glucometer as per manufacturer instruction using blood from the right atrium taken during non-survival tissue collection.

### Pathology Core Work-Up

Histological analysis was performed by Vanderbilt Pathology Core. Four male P17 mice (2 Dlx5 KCC2 cKO, 2 WT siblings) were analyzed grossly, histologically, and by complete blood count. Mice were euthanized with carbon dioxide. Blood was collected via intracardiac puncture and placed in an EDTA tube. Complete blood count was performed on the Forcyte Hematology Analyzer (Oxford Science, Oxford, CT United States). Gross necropsy was performed, and a complete set of tissues were collected and submerged in 10% neutral buffered formalin for 48 h fixation. Tissues were processed routinely, embedded in paraffin, sectioned at 5 microns and stained with hematoxylin and eosin. Additionally, sections of the brain were stained with Fluro-Jade.

### Tissue Collection and Preparation

Tissue for immunohistochemistry and fluorophore imaging was collected and fixed either by brief or heavy fixation. For brief fixation, mice were anesthetized, and brains were dissected and immersed in 4% paraformaldehyde (PFA) for 15 min. For heavy fixation, mice were anesthetized, transcardially perfused with PBS, then 4% PFA. The brains were then dissected and immersed in 4% PFA overnight at 4°C. After brief or heavy fixation, brains were washed in PBS, transferred to 30% sucrose solution for a week at 4°C for cryoprotection, blocked, immersed in OCT compound, and flash-frozen in liquid nitrogen. Coronal sections containing barrel cortex were sectioned at 20 μm on a Leica cryostat and stored at −80°C.

### Immunohistochemistry

For most applications, we used briefly fixed tissue, but heavily fixed tissue was used for parvalbumin and sometimes somatostatin staining with inclusion of comparable controls. The slides were dried for 30 min, then incubated in 4% PFA for 5 min. For heavily fixed tissue only, there were then two PBS washes, and unmasking for 30 min in 50 mM Na-Citrate at 80°C. Both briefly and heavily fixed tissue was then washed (PBS + 0.1% Triton X-100), blocked overnight at 4°C in blocking solution (PBS + 0.5% Triton X-100 + 8% horse serum), washed, incubated overnight at 4°C in primary antibody (Table 1) in blocking solution, washed, incubated overnight at 4°C in secondary antibody (Table 2) in blocking solution, washed, dried, and mounted/coverslipped in hard-setting Vectashield or Prolong Gold mounting medium with DAPI. Sections were allowed to cure for at least 30 min, then stored at 4°C and imaged within 2 weeks. When visualizing fluorophores without immunohistochemistry, sections were thawed and mounted/coverslipped.

**TABLE 1:** List of primary antibodies used.

Target	Host	Vendor name	Catalog #	Dilution	Type
KCC2	Rb	EMD-Millipore/Upstate	07-432	1:250	IgG
Parvalbumin	Ms	Sigma	P3088	1:250	IgG
Somatostatin	Rb	Santa Cruz	Sc-13099	1:250	IgG
vGAT	Gp	SYSY	131-004	1:250	IgG
vGlut2	Ms	Neuromab/UC Davis	75-067	1:500	IgG
Gephyrin	Ms	SYSY	147021	1:250	IgG_1K_
α1 GABA-A receptor	Rb	Millipore	06-868	1:500	IgG
α2 GABA-A receptor	Rb	Abcam	ab72445	1:250	IgG
α3 GABA-A receptor	Rb	Alomone	AGA-003	1:500	IgG
α4 GABA-A receptor	Rb	Novus	NB300-194	1:500	IgG
α5 GABA-A receptor	Rb	Millipore	AB9678	1:250	IgG
β2 GABA-A receptor	Rb	Millipore	AB5561	1:250	IgG
β3 GABA-A receptor	Rb	Novus	NB300-199	1:250	IgG
δ GABA-A receptor	Rb	R&D Systems	PPS090	1:250	IgG
γ2 GABA-A receptor	Rb	Synaptic Systems	224-003	1:250	IgG

*Host species abbreviations are: Rb, rabbit; Ms, mouse; Gp, Guinea Pig.*

**TABLE 2:** List of secondary antibodies used.

Target	Fluorophore	Host	Vendor name	Catalog #	Dilution
Anti-Rabbit IgG (H + L)	Cy3	Donkey	Jackson ImmunoResearch	711-165-152	1:250-1000
Anti-Rabbit IgG (H + L) anti-Mouse	Cy5	Donkey	Jackson ImmunoResearch	711-165-152	1:100-500
Anti-Mouse IgG (H + L)	Cy3	Donkey	Jackson ImmunoResearch	715-165-150	1:250-500
Anti-Mouse (H + L)	Alexa-647	Donkey	Jackson ImmunoResearch	715-605-150	1:250
Anti-Mouse (H + L)	Cy5	Donkey	Jackson ImmunoResearch	715-605-150	1:100-250
Anti-Guinea Pig IgG (H + L)	Alexa-488	Donkey	Jackson ImmunoResearch	706-545-148	1:100-250
Anti-Guinea Pig IgG (H + L)	Cy3	Donkey	Jackson ImmunoResearch	706-165-148	1:100-250

### EdU-Labeling Interneurons

Pregnant mouse dams were injected at E13.5 with 1 mg EdU/10 g body weight, delivered as 10 mg/mL solution of 5-Ethynyl-2′-deoxyuridine (EdU, from Carbosynth) in 0.9% sodium chloride saline. Brain tissue was collected from P0 pups by anesthetizing pups, decapitation, 30 min immersion of the head in 4% PFA, and further processing/cryosectioning as outlined earlier in the protocol. To label EdU, slide-mounted sections were rinsed in PBS thrice (rinsed), permeabilized in PBS + 0.5% Triton X-100, rinsed, incubated for 5 min in CLICK reaction cocktail [4 mM CuSO_4_ pentahydrate, 5 μM Sulfo-Cyanine 5 Azide (Lumiprobe #A3330), 100 mM sodium ascorbate in Tris-buffered saline], rinsed, dried for 30 min, and mounted/coverslipped using Prolong Gold with DAPI.

### Image Acquisition and Analysis

Brain sections were imaged using a Leica DM 6000 epifluorescent microscope equipped with a DFC365 FX digital camera and a computer running Leica LAS X software (Leica, Buffalo Grove, IL, United States). Images were acquired using 5 and 10× objectives. Images were stitched using Fiji ImageJ software stitching plugin (Preibisch et al., 2009): stitching-grid/collection stitching, 30% overlap, maximum intensity fusion method with subpixel accuracy. The resulting fused images were saved as 8-bit tagged image files (TIFs). Confocal images were taken using a Leica TCS SPE/DM2500 microscope with 63× oil immersion objective and a computer running Leica LAS software.

Image analysis and editing was done using Fiji ImageJ. For immunohistochemistry images that compare expression intensity, images were acquired with similar settings and minimally edited. For KCC2/reporter co-localization, projections from confocal image stacks were made using Fiji ImageJ. Brain regions were identified using Paxinos (2007) and Schambra and Schambra (2008). When needed, marginal zone and subplate were further identified using Bayer and Altman (1990) and Qu et al. (2016). Cortical layers were defined using DAPI staining of our tissue. For separate analysis, these regions were segregated into regions of interest (ROIs). EdU+, TdTomato+ cells, PV+ cells, and SST+ cells were counted manually using the cell counter plugin. Counts of TdTomato+ cells and VGAT+ particles were done by manually setting a threshold for each image that distinguished cells/particles from background, and then automatically counting particles with the “analyze particles” feature. These automatic IN counts were compared to be consistent with manual counts. Density was calculated as number of cells/particles divided by ROI area.

### Electrophysiology

P12–P14 mice were anesthetized and transcardially perfused with cold, oxygenated cutting solution, consisting of: 200 mM sucrose, 1.9 mM KCl, 1.2 mM NaH_2_PO_4_, 6 mM MgCl_2_, 0.5 mM CaCl_2_, 10 mM glucose, 25 mM NaHCO_3_ (305 mOsm, pH 7.4). Mice were then decapitated, their brains were dissected, blocked, and coronal slices were sectioned at 300 μm on a Leica VT1200S vibratome in cold, oxygenated cutting solution. Slices were then recovered for 30 min at 34°C and 1 hr at room temperature (20–21°C) in aCSF, consisting of: 125 mM NaCl, 2.5 mM KCl, 1.25 mM NaH_2_PO_4_, 1.3 mM MgCl_2_, 0.2 mM CaCl_2_, 10 mM glucose, 25 mM NaHCO_3_ (305 mOsm, pH 7.4). Recordings were performed in a chamber perfused with 20–21°C oxygenated aCSF, perfused at a rate of 2.0 mL/min. Pyramidal neurons of layer 5 of barrel field cortex was targeted based on anatomical location, visible barrels, and cellular morphology. Neurons were patched with 3–5 MΩ glass electrodes filled with internal solution containing: 150 mM CsCl, 1 mM MgCl_2_, 10 mM HEPES, 0.1 mM CaCl_2_, 1.1 mM EGTA, and 2 mM Na_2_ATP (285 mOsm, pH 7.4) and clamped at −70 mV. Whole-cell recordings were acquired using an Axon MultiClamp 700B amplifier, filtered at 2 kHz, digitized at 10 kHz with Digidata 1440A, and recorded with ClampeX 10.4 software. sIPSCs were recorded with 10 μM NBQX and 50 μM APV in aCSF and analyzed with miniAnalysis software with event threshold set at 15 pA. Series resistance (*R*_a_) was uncorrected, and recordings were discarded if *R*_a_ was over 25 MΩ or changed by more than 15% over the course of a recording. For IPSC kinetics analysis, events with a rise time of more than 3 ms were discarded due to space-clamp concerns. Medians of amplitude, rise time, decay, area, and interevent interval were calculated for each cell and used for WT vs. Dlx5 KCC2 cKO comparison.

### Flurothyl Seizure Induction

Mice were placed individually into an airtight, clear acrylic chamber with internal volume of 2 L. A 10% (v/v) solution of flurothyl (bis-2,2,2-trifluoroethyl ether, Sigma-Aldrich) dissolved in 95% ethanol was delivered via precision syringe pump at a rate of 100 μL/min through a port in the chamber lid onto an absorbent pad in a tray suspended from the underside of the lid. The onset of seizure was defined as the point at which the mouse started convulsing with loss of postural control. The time latency (in seconds) to the onset of seizure was measured from the first drop of flurothyl onto the filter paper. The chamber was vented and cleansed of all flurothyl residue between trials. All flurothyl trials were video recorded for later review. The experimenter remained blinded to animal genotypes throughout all phases of data acquisition.

### Video Monitoring

Cages of dams with litters containing Dlx5 KCC2 cKO pups were nightly monitored for seizure activity using an infrared DVC 24.0 megapixel HD camera at 720 p/30 fps and VGA/30 fps settings, positioned in front of the cage in the mouse housing facility. Whenever a pup died, we reviewed the previous night’s recordings, looking for pups venturing from the nest and convulsing. Litters were checked daily for dead pups. The camera captured the entirety of the cage, but pups in the nest or buried in bedding were occluded from view. WT littermates were kept in the cages with Dlx5 KCC2 cKO to keep conditions as close to normal, and minimize stress and subsequent seizure provocation. Dlx5 KCC2 cKO pups were identified by their smaller size. Unlike Dlx5 KCC2 cKO pups, WT pups seldom left the nest in the first two postnatal weeks, which made it easy to observe the Dlx5 KCC2 cKO. Seizure activity was judged based on observable clonus.

### Data Analysis and Statistics

All numerical data was stored in data tables using Microsoft Excel 2016. Statistical analysis and graph preparation was done in GraphPad Prism 7.0. All bar graphs display an average with standard error of the mean. Statistical tests and graph types used are described in the legend of each figure.

## Data Availability Statement

The raw data supporting the conclusions of this article will be made available by the authors, without undue reservation.

## Ethics Statement

The animal study was reviewed and approved by Vanderbilt’s Institutional Animal Care and Use Committee.

## Author Contributions

KZ, ED, and AL contributed to the conception and design of the study. ED contributed the KCC2 flox mouse, which he created. KZ did most of the experiments, performed statistical analysis, made most of the figures, and wrote the first draft of the manuscript, which was significantly revised together with AL. AH helped with designing and conducting histology experiments and animal husbandry and molecular biology for this project, optimized and performed GABAA receptor subunit and VGluT2 immunostaining experiments, and made Supplementary Figure 3. CF contributed as a collaborator by conceiving, designing, and performing the flurothyl seizure experiments and Nkx2.1 Dlx5 cKO survival experiments, as well as doing statistical analysis, figures, and writing methods for these sections (panels in Figures 4, 7). All authors contributed to manuscript revision, read, and approved the submitted version.

## Conflict of Interest

The authors declare that the research was conducted in the absence of any commercial or financial relationships that could be construed as a potential conflict of interest.

## Publisher’s Note

All claims expressed in this article are solely those of the authors and do not necessarily represent those of their affiliated organizations, or those of the publisher, the editors and the reviewers. Any product that may be evaluated in this article, or claim that may be made by its manufacturer, is not guaranteed or endorsed by the publisher.
