# Supplementary material for: Loss of KCC2 in GABAergic Neurons Causes Seizures and an Imbalance of Cortical Interneurons
Source: Front Mol Neurosci. 2022 Mar 16;15:826427. doi: 10.3389/fnmol.2022.826427 (PMC8966887; doi:10.3389/fnmol.2022.826427)
Supplement: Supplementary file 1 [file Data_Sheet_1.PDF]

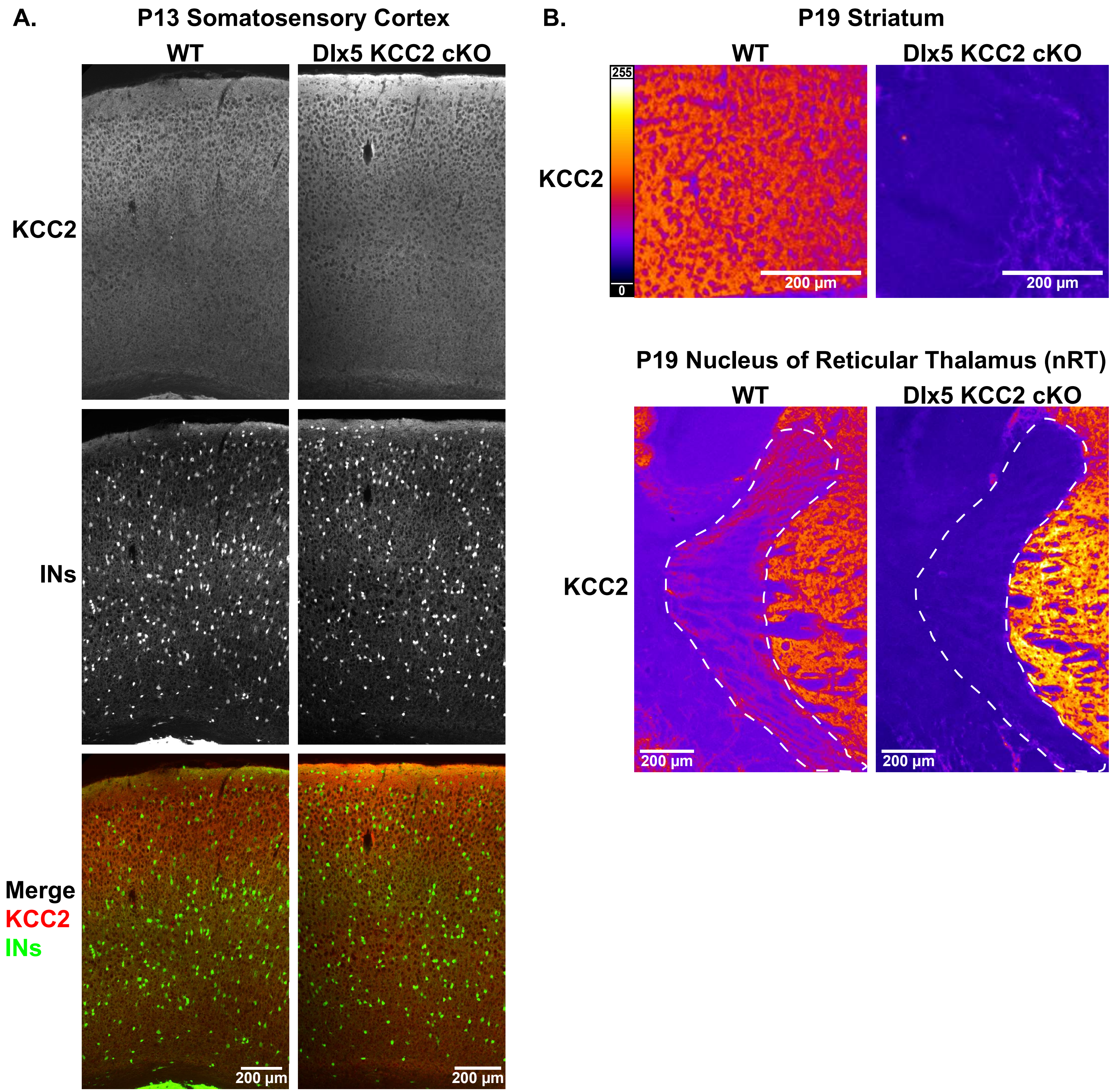


**Supplementary Figure 1. Close-ups of KCC2 expression in Dlx5 KCC2 cKO P13 cortex, and P19 striatum and nRT.** (A) 10x-acquired images of KCC2 immunoreactivity and tdTomato-labeled interneurons (INs) in P13 somatosensory cortex of Dlx5 KCC2 cKO and WT control. Note comparable KCC2 expression due to a large volume of KCC2-expressing cells not affected by the cKO. (B) Close-ups of 5x-acquired images of KCC2 immunoreactivity in striatum and nRT of Dlx5 KCC2 cKO and WT control at P19, shown in heatmap. nRT is traced with a white dashed outline. Notice a near-complete lack of KCC2 immunoreactivity in nRT and striatum of Dlx5 KCC2 cKO.
