# Supplementary material for: Loss of KCC2 in GABAergic Neurons Causes Seizures and an Imbalance of Cortical Interneurons
Source: Front Mol Neurosci. 2022 Mar 16;15:826427. doi: 10.3389/fnmol.2022.826427 (PMC8966887; doi:10.3389/fnmol.2022.826427)
Supplement: Supplementary file 2 [file Data_Sheet_2.PDF]

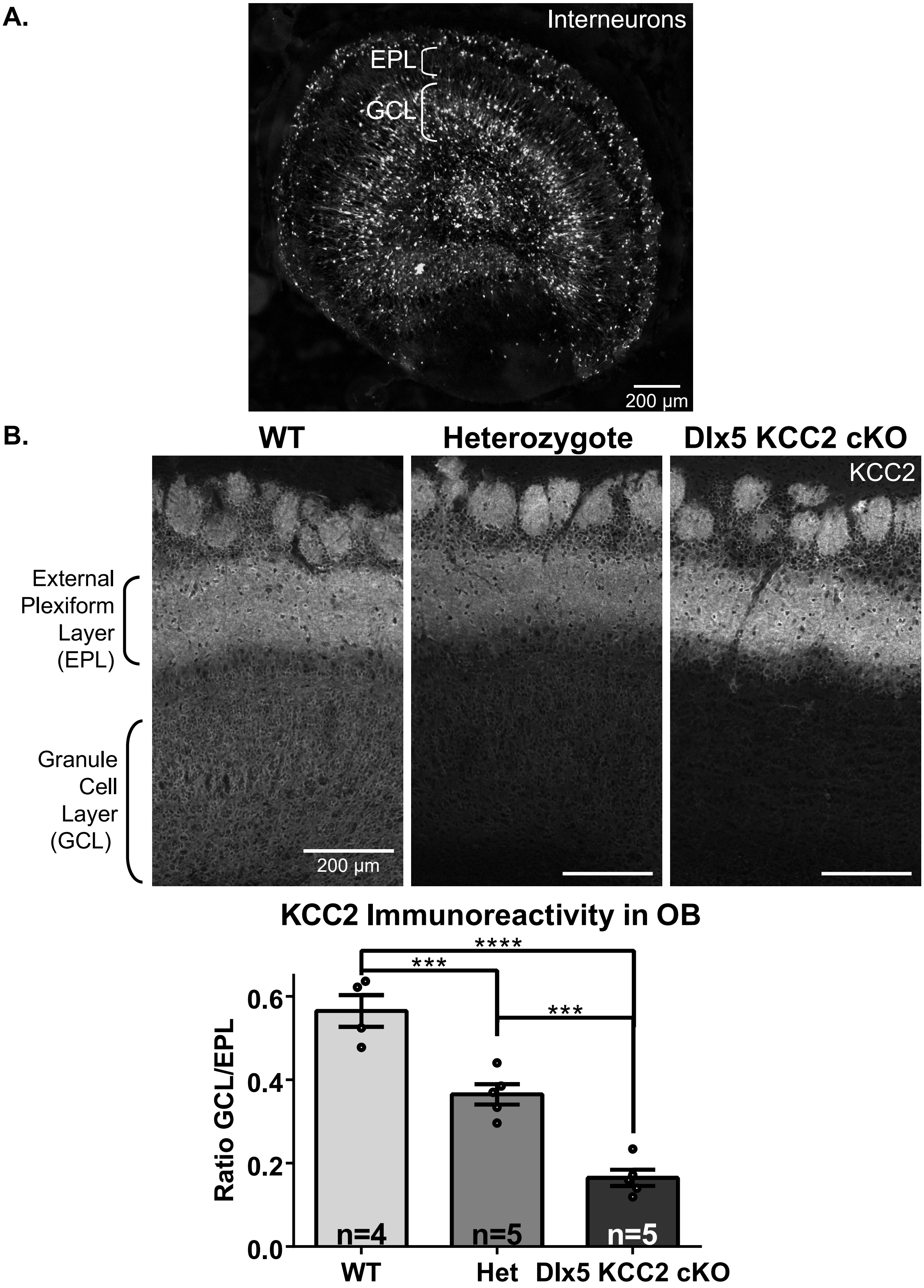

Supplementary Figure 2. Dlx5 KCC2 cKO and heterozygotes have reduced KCC2 expression in olfactory bulb GCL. (A) Interneurons in the olfactory bulb (OB) of P6 heterozygote marked by Dlx5-driven GFP. The granule cell layer (GCL) is particularly rich in Dlx5-lineage neurons, while the external plexiform layer (EPL) has few interneurons. (B) We confirmed targeted loss of KCC2 expression in the Dlx5 KCC2 cKO by greatly reduced KCC2 immunoreactivity within GCL at P18-20 (images). We quantified this reduction by taking a ratio of intensities of GCL and EPL (bar graph). Note that the heterozygote also shows a considerable reduction in KCC2 immunoreactivity. One-way ANOVA with Tukey's multiple comparisons test: ****P<.0001, ***P<.001. n= 4 wildtype sibling (WT), 5 heterozygote sibling (Het), 5 Dlx5 KCC2 cKO mice, 3 images/mouse.
