# Supplementary material for: Loss of KCC2 in GABAergic Neurons Causes Seizures and an Imbalance of Cortical Interneurons
Source: Front Mol Neurosci. 2022 Mar 16;15:826427. doi: 10.3389/fnmol.2022.826427 (PMC8966887; doi:10.3389/fnmol.2022.826427)
Supplement: Supplementary file 3 [file Data_Sheet_3.pdf]

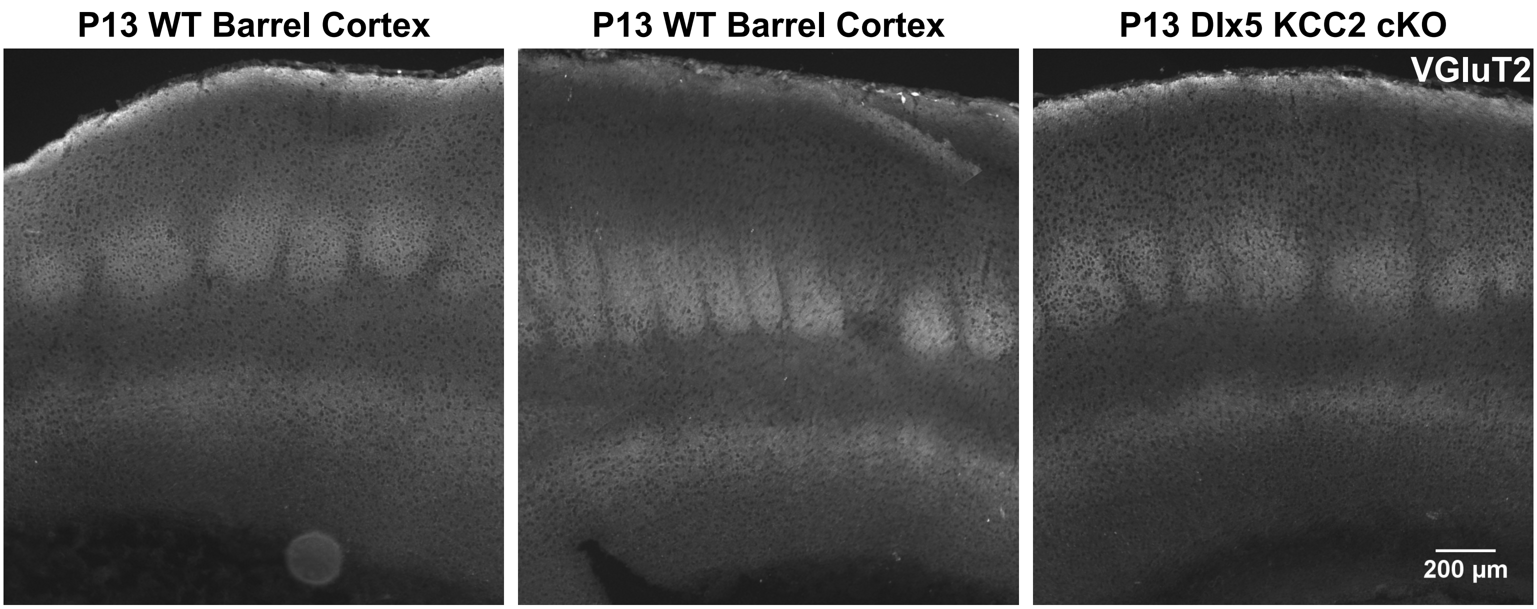

Supplementary Figure 3. Normal thalamocortical projections in Dlx5 KCC2 cKO. Images show VGluT2 immunostaining, which marks presynaptic terminals of thalamocortical afferents, in P13 barrel cortex of WT and Dlx5 KCC2 cKO.
