# Supplementary material for: Loss of KCC2 in GABAergic Neurons Causes Seizures and an Imbalance of Cortical Interneurons
Source: Front Mol Neurosci. 2022 Mar 16;15:826427. doi: 10.3389/fnmol.2022.826427 (PMC8966887; doi:10.3389/fnmol.2022.826427)
Supplement: Supplementary file 4 [file Data_Sheet_4.PDF]

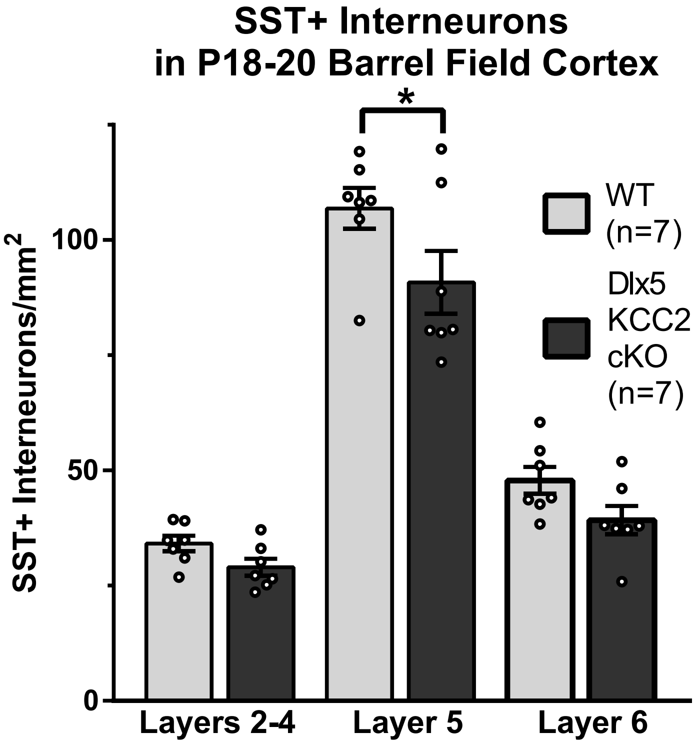

Supplementary Figure 4. Decrease in layer 5 SST+ INs in P18-20 Dlx5 KCC2 cKO. Bar graph shows densities of SST+ INs by layers in P18-20 barrel field cortex. Note that only a subset of Dlx5 KCC2 cKO mice survive to the P18-20 timepoint. *P<.05 by one-way ANOVA with Sidak's multiple comparisons test.
