# Supplementary material for: Loss of KCC2 in GABAergic Neurons Causes Seizures and an Imbalance of Cortical Interneurons
Source: Front Mol Neurosci. 2022 Mar 16;15:826427. doi: 10.3389/fnmol.2022.826427 (PMC8966887; doi:10.3389/fnmol.2022.826427)
Supplement: Supplementary file 5 [file Data_Sheet_5.PDF]

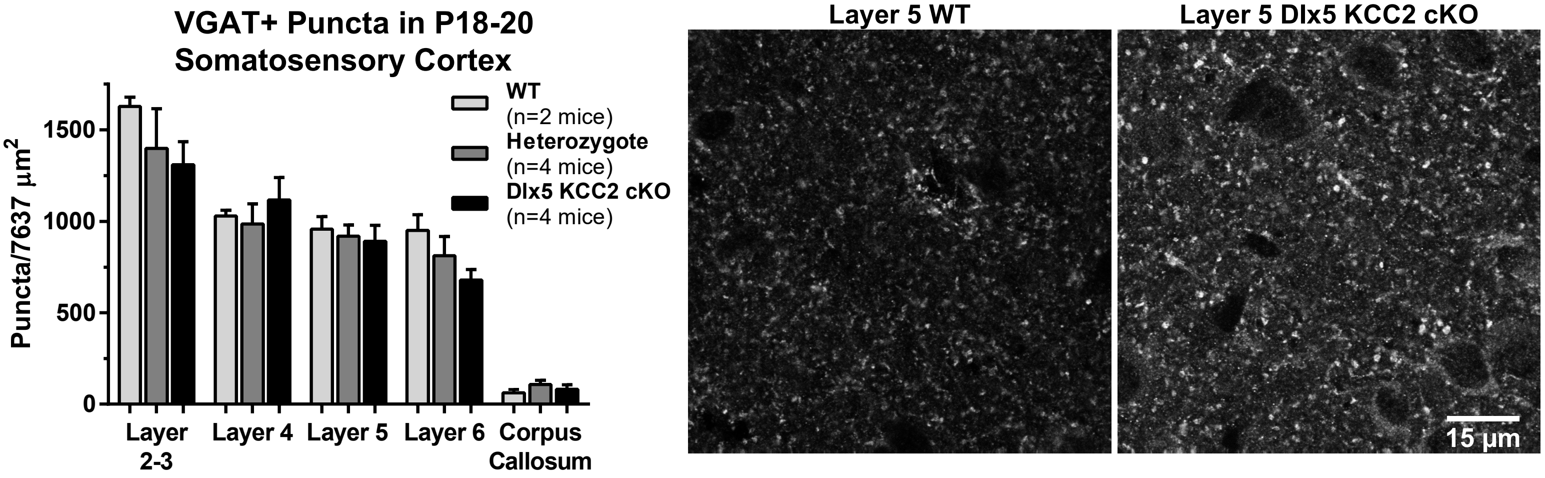

Supplementary Figure 5. Normal density of inhibitory synapses in Dlx5 KCC2 cKO cortex. Bar graph shows density of VGAT+ puncta, which mark inhibitory presynaptic terminals, in different layers of somatosensory cortex of P18-20 Dlx5 KCC2 cKO, sibling heterozygotes, and sibling WT controls. Images on the right show examples of VGAT staining in layer 5 of WT and Dlx5 KCC2 cKO. No significant differences were found by one-way ANOVA with post-hoc Sidak’s multiple comparisons test.
