# Supplementary material for: Loss of KCC2 in GABAergic Neurons Causes Seizures and an Imbalance of Cortical Interneurons
Source: Front Mol Neurosci. 2022 Mar 16;15:826427. doi: 10.3389/fnmol.2022.826427 (PMC8966887; doi:10.3389/fnmol.2022.826427)
Supplement: Supplementary file 6 [file Data_Sheet_6.PDF]

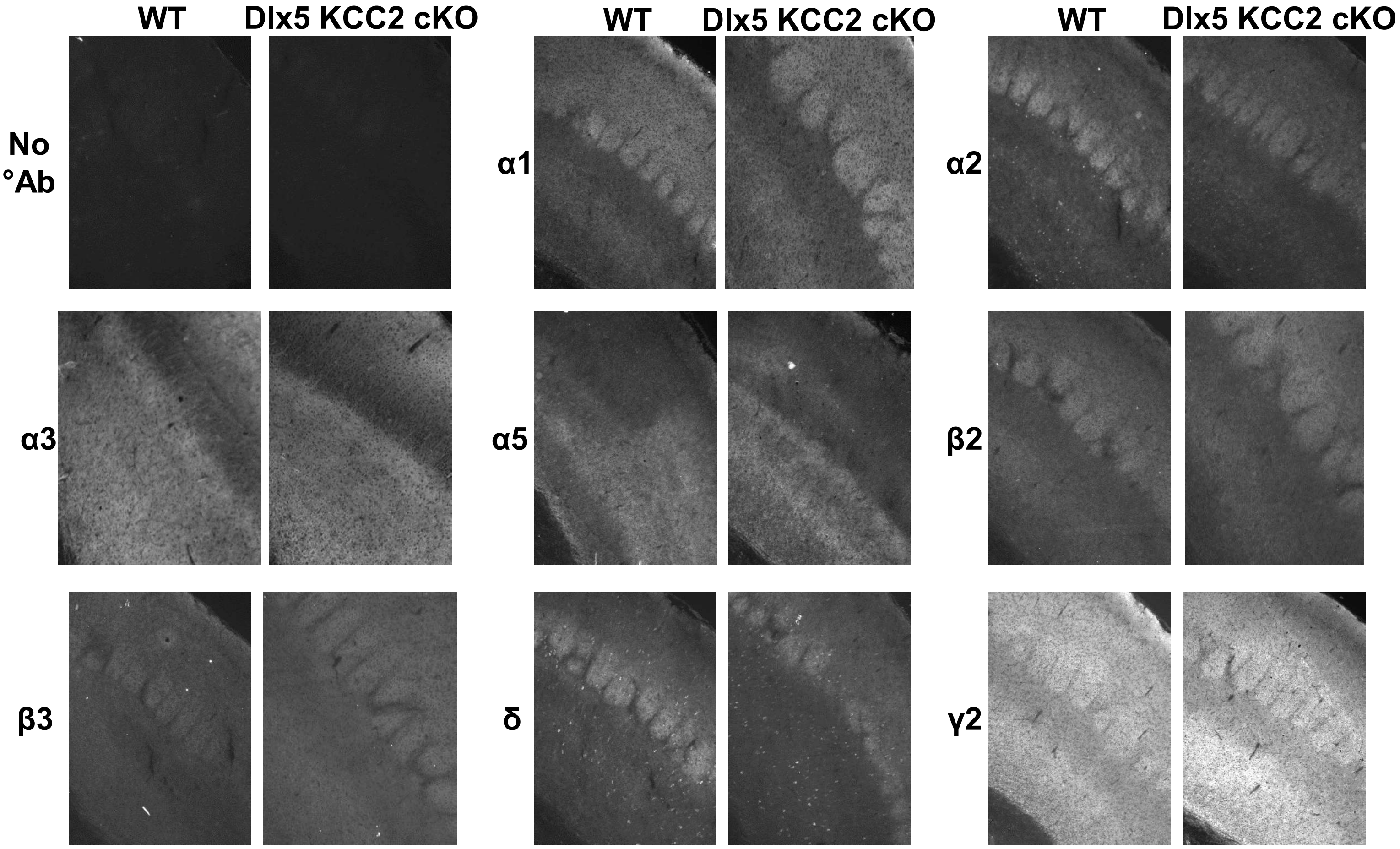

Supplementary Figure 6. Normal GABAA receptor subunit expression in Dlx5 KCC2 cKO cortex. Images show immunohistochemical staining of barrel cortex of P12-14 Dlx5 KCC2 cKO and sibling WT for different subunits of GABAA receptors, indicated on the left of each image pair. Cortical surface/layer 1 is in top right corner of each image. Staining without a primary antibody is provided in top left to indicate the level of off-target immunoreactivity.
